# 3-Acetyl-1-(2,3-dimethyl­phen­yl)thio­urea

**DOI:** 10.1107/S1600536812027973

**Published:** 2012-06-23

**Authors:** Sharatha Kumar, Sabine Foro, B. Thimme Gowda

**Affiliations:** a Department of Chemistry, Mangalore University, Mangalagangotri 574 199, Mangalore, India; b Institute of Materials Science, Darmstadt University of Technology, Petersenstrasse 23, D-64287 Darmstadt, Germany

## Abstract

In the crystal structure of the title compound, C_11_H_14_N_2_OS, the conformation of the two N—H bonds is *anti*. The conformation of the C=S and the C=O bonds is also *anti*. Furthermore, the N—H bond adjacent to the benzene ring is *anti* to the *ortho*- and *meta*-methyl groups. The dihedral angle between the benzene ring and the side chain [N—C(= S)—N—C(=O)—C; maximum deviation = 0.047 (4) Å] is 81.33 (10)°. The NH hydrogen adjacent to the benzene ring and the amide O atom exhibit bifurcated intra- and inter­molecular hydrogen bonding. In the crystal, mol­ecules form inversion dimers, which are linked into chains *via R*_2_^2^(12) and *R*_2_^2^(8) networks.

## Related literature

For studies on the effects of substituents on the structures and other aspects of *N*-(ar­yl)-amides, see: Bhat & Gowda (2000 ▶); Gowda *et al.* (2006 ▶); Shahwar *et al.* (2012 ▶), of *N*-(ar­yl)-methane­sulfonamides, see: Gowda *et al.* (2007 ▶) and of *N*-chloro­aryl­sulfonamides, see: Jyothi & Gowda (2004 ▶); Shetty & Gowda (2004 ▶).
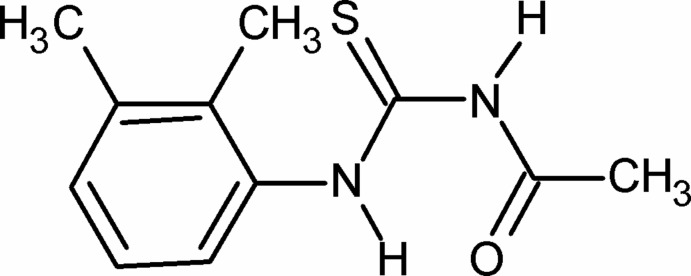


## Experimental

### 

#### Crystal data

C_11_H_14_N_2_OS
*M* *_r_* = 222.30
Triclinic, 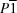

*a* = 5.0552 (7) Å
*b* = 9.869 (2) Å
*c* = 12.028 (3) Å
α = 106.71 (1)°
β = 91.01 (1)°
γ = 94.57 (1)°
*V* = 572.4 (2) Å^3^
*Z* = 2
Mo *K*α radiation
μ = 0.26 mm^−1^
*T* = 293 K
0.48 × 0.08 × 0.04 mm

#### Data collection

Oxford Diffraction Xcalibur diffractometer with a Sapphire CCD detector
Absorption correction: multi-scan (*CrysAlis RED*; Oxford Diffraction, 2009 ▶) *T*_min_ = 0.886, *T*_max_ = 0.990
3414 measured reflections
2066 independent reflections
1331 reflections with *I* > 2σ(*I*)
*R*_int_ = 0.028

#### Refinement

*R*[*F* ^2^ > 2σ(*F* ^2^)] = 0.065
*wR*(*F* ^2^) = 0.141
*S* = 1.08
2066 reflections
145 parameters
5 restraints
H atoms treated by a mixture of independent and constrained refinement
Δρ_max_ = 0.33 e Å^−3^
Δρ_min_ = −0.24 e Å^−3^

### 

Data collection: *CrysAlis CCD* (Oxford Diffraction, 2009 ▶); cell refinement: *CrysAlis CCD*; data reduction: *CrysAlis RED* (Oxford Diffraction, 2009 ▶); program(s) used to solve structure: *SHELXS97* (Sheldrick, 2008 ▶); program(s) used to refine structure: *SHELXL97* (Sheldrick, 2008 ▶); molecular graphics: *PLATON* (Spek, 2009 ▶); software used to prepare material for publication: *SHELXL97*.

## Supplementary Material

Crystal structure: contains datablock(s) I, global. DOI: 10.1107/S1600536812027973/nc2285sup1.cif

Structure factors: contains datablock(s) I. DOI: 10.1107/S1600536812027973/nc2285Isup2.hkl

Supplementary material file. DOI: 10.1107/S1600536812027973/nc2285Isup3.cml

Additional supplementary materials:  crystallographic information; 3D view; checkCIF report

## Figures and Tables

**Table 1:** Hydrogen-bond geometry (Å, °)

*D*—H⋯*A*	*D*—H	H⋯*A*	*D*⋯*A*	*D*—H⋯*A*
N1—H1*N*⋯O1	0.86 (2)	1.97 (3)	2.664 (4)	137 (3)
N1—H1*N*⋯O1^i^	0.86 (2)	2.50 (3)	3.168 (4)	136 (3)
N2—H2*N*⋯S1^ii^	0.84 (2)	2.54 (2)	3.378 (3)	176 (3)

Symmetry codes: (i) 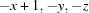; (ii) 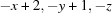.

**Fig. 1.**
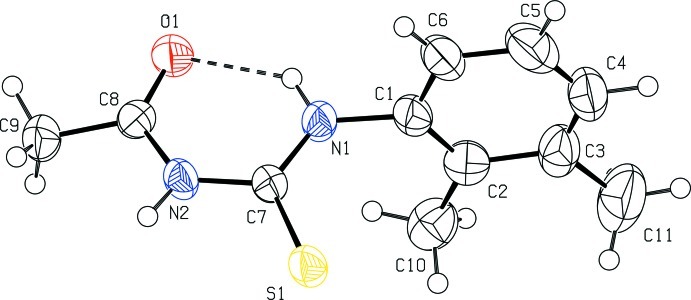
Molecular structure of the title compound, showing the atom labelling scheme and with displacement ellipsoids drawn at the 50% probability level.

**Fig. 2.**
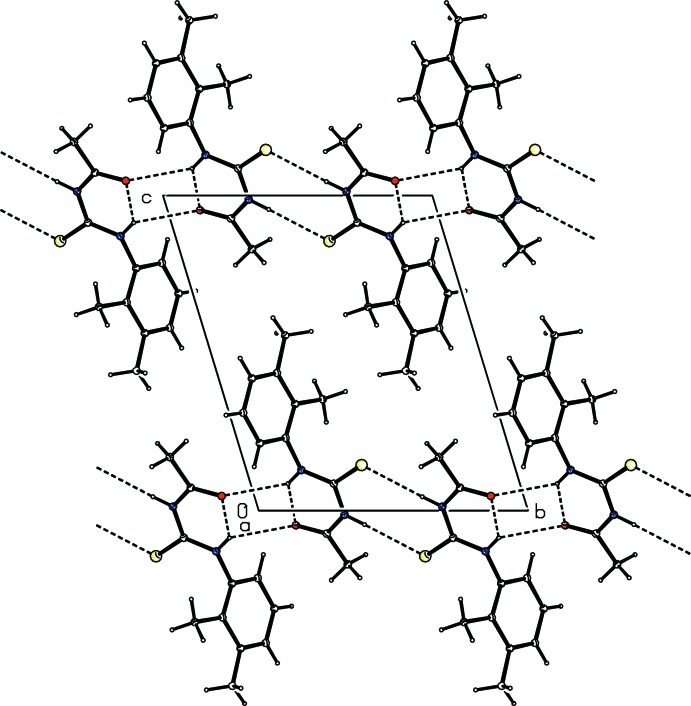
Molecular packing of the title compound with hydrogen bonding shown as dashed lines.

## supplementary crystallographic information

### Comment

Thiourea and its derivatives are widely used as precursors or intermediates in synthetic organic chemistry. They are known to exhibit a wide variety of biological activities. As part of our studies on the substituent effects on the structures and other aspects of *N*-(aryl)-amides (Bhat & Gowda, 2000; Gowda *et al.*, 2006; Shahwar *et al.*, 2012); *N*-(aryl)-methanesulfonamides (Gowda *et al.*, 2007) and *N*-chloroarylsulfonamides (Jyothi & Gowda, 2004; Shetty & Gowda, 2004), in the present work, the crystal structure of 3-acetyl-1-(2,3-dimethylphenyl)thiourea has been determined (Fig. 1).

The conformation of the two N—H bonds are *anti* to each other, and one of them is *anti* to the C═S in the urea segment and the other orients away from it. The adjacent N—H bond is *anti* to the *ortho*- and *meta*-methyl groups in the benzene ring. Furthermore, the conformations of the amide C═S and the C═O are *anti* to each other, similar to the *anti* conformation observed in 3-acetyl-1-(2-methylphenyl)thiourea (Shahwar *et al.*, 2012).

The side chain is oriented itself with respect to the phenyl ring with the torsion angles of C2—C1—N1—C7 = 83.59 (47)° and C6—C1—N1—C7 = - 99.89 (44)°. The dihedral angle between the phenyl ring and the side chain is 81.33 (10)°.

The hydrogen atom of the NH attached to the phenyl ring and the amide oxygen exhibit a bifurcated hydrogen bonding by showing the simultaneous intra and intermolecular hydrogen bonding. In the crystal, the molecules form inversion type dimers which are linked into infinite chains in terms of *R*_2_^2^(12) and *R*_2_^2^(8) networks through series of N—H···O and N—H···S intermolecular hydrogen bonds, respectively (Table 1, Fig.2).

### Experimental

3-Acetyl-1-(2,3-dimethylphenyl)thiourea was synthesized by adding a solution of acetyl chloride (0.10 mol) in acetone (30 ml) dropwise to a suspension of ammonium thiocyanate (0.10 mol) in acetone (30 ml). The reaction mixture was refluxed for 30 min. After cooling to room temperature, a solution of 2,3-dimethylaniline (0.10 mol) in acetone (10 ml) was added and refluxed for 3 h. The reaction mixture was poured into acidified cold water. The precipitated title compound was recrystallized to constant melting point from acetonitrile. The purity of the compound was checked and characterized by its infrared spectrum.

Needle like colourless single crystals used in X-ray diffraction studies were grown in acetonitrile solution by slow evaporation of the solvent at room temperature.

### Refinement

All C—H H atoms were positioned with idealized geometry (methyl H atoms allowed to rotate but not to tip) and were refined isotropic with *U*_iso_(H) = 1.2 *U*_eq_(C) (1.5 for methyl H atoms) using a riding model with C—H = 0.93 Å for aromatic and C—H = 0.96 Å for methyl H atoms. The amino H atoms were refined with the N—H distances restrained to 0.86 (2) Å.

### Crystal data

**Table:**

C_11_H_14_N_2_OS	*Z* = 2
*M**_r_* = 222.30	*F*(000) = 236
Triclinic, *P*1	*D*_x_ = 1.290 Mg m^−^^3^
Hall symbol: -P 1	Mo *K*α radiation, λ = 0.71073 Å
*a* = 5.0552 (7) Å	Cell parameters from 1147 reflections
*b* = 9.869 (2) Å	θ = 3.5–27.8°
*c* = 12.028 (3) Å	µ = 0.26 mm^−^^1^
α = 106.71 (1)°	*T* = 293 K
β = 91.01 (1)°	Needle, colourless
γ = 94.57 (1)°	0.48 × 0.08 × 0.04 mm
*V* = 572.4 (2) Å^3^	

### Data collection

**Table:**

Oxford Diffraction Xcalibur diffractometer with a Sapphire CCD detector	2066 independent reflections
Radiation source: fine-focus sealed tube	1331 reflections with *I* > 2σ(*I*)
Graphite monochromator	*R*_int_ = 0.028
Rotation method data acquisition using ω and phi scans	θ_max_ = 25.3°, θ_min_ = 3.5°
Absorption correction: multi-scan (*CrysAlis RED*; Oxford Diffraction, 2009)	*h* = −6→6
*T*_min_ = 0.886, *T*_max_ = 0.990	*k* = −11→11
3414 measured reflections	*l* = −13→14

### Refinement

**Table:**

Refinement on *F*^2^	Primary atom site location: structure-invariant direct methods
Least-squares matrix: full	Secondary atom site location: difference Fourier map
*R*[*F*^2^ > 2σ(*F*^2^)] = 0.065	Hydrogen site location: inferred from neighbouring sites
*wR*(*F*^2^) = 0.141	H atoms treated by a mixture of independent and constrained refinement
*S* = 1.08	*w* = 1/[σ^2^(*F*_o_^2^) + (0.0493*P*)^2^ + 0.4018*P*] where *P* = (*F*_o_^2^ + 2*F*_c_^2^)/3
2066 reflections	(Δ/σ)_max_ = 0.003
145 parameters	Δρ_max_ = 0.33 e Å^−^^3^
5 restraints	Δρ_min_ = −0.24 e Å^−^^3^

### Special details

**Table:**

Experimental. Absorption correction: CrysAlis RED (Oxford Diffraction, 2009) Empirical absorption correction using spherical harmonics, implemented in SCALE3 ABSPACK scaling algorithm.
Geometry. All e.s.d.'s (except the e.s.d. in the dihedral angle between two l.s. planes) are estimated using the full covariance matrix. The cell e.s.d.'s are taken into account individually in the estimation of e.s.d.'s in distances, angles and torsion angles; correlations between e.s.d.'s in cell parameters are only used when they are defined by crystal symmetry. An approximate (isotropic) treatment of cell e.s.d.'s is used for estimating e.s.d.'s involving l.s. planes.
Refinement. Refinement of *F*^2^ against ALL reflections. The weighted *R*-factor *wR* and goodness of fit *S* are based on *F*^2^, conventional *R*-factors *R* are based on *F*, with *F* set to zero for negative *F*^2^. The threshold expression of *F*^2^ > σ(*F*^2^) is used only for calculating *R*-factors(gt) *etc*. and is not relevant to the choice of reflections for refinement. *R*-factors based on *F*^2^ are statistically about twice as large as those based on *F*, and *R*- factors based on ALL data will be even larger.

### Fractional atomic coordinates and isotropic or equivalent isotropic displacement parameters (Å^2^)

**Table:**

	*x*	*y*	*z*	*U*_iso_*/*U*_eq_	
S1	1.1098 (2)	0.43318 (11)	0.14467 (9)	0.0458 (3)	
O1	0.4254 (5)	0.1213 (3)	−0.0465 (2)	0.0552 (8)	
N1	0.7872 (6)	0.2041 (3)	0.1289 (3)	0.0432 (8)	
H1N	0.674 (6)	0.140 (3)	0.086 (3)	0.052*	
N2	0.7252 (6)	0.3141 (3)	−0.0129 (3)	0.0382 (7)	
H2N	0.773 (7)	0.378 (3)	−0.043 (3)	0.046*	
C1	0.9070 (7)	0.1817 (4)	0.2310 (3)	0.0438 (10)	
C2	0.8249 (7)	0.2551 (4)	0.3383 (3)	0.0477 (10)	
C3	0.9350 (9)	0.2228 (5)	0.4369 (4)	0.0584 (10)	
C4	1.1149 (9)	0.1232 (5)	0.4189 (4)	0.0658 (11)	
H4	1.1871	0.1023	0.4831	0.079*	
C5	1.1947 (10)	0.0521 (5)	0.3101 (4)	0.0717 (12)	
H5	1.3189	−0.0143	0.3020	0.086*	
C6	1.0896 (8)	0.0800 (4)	0.2137 (4)	0.0538 (11)	
H6	1.1389	0.0326	0.1394	0.065*	
C7	0.8621 (7)	0.3091 (4)	0.0861 (3)	0.0356 (8)	
C8	0.5121 (7)	0.2265 (4)	−0.0728 (3)	0.0384 (9)	
C9	0.3956 (8)	0.2699 (4)	−0.1700 (3)	0.0509 (10)	
H9A	0.5356	0.2990	−0.2134	0.076*	
H9B	0.2895	0.3476	−0.1395	0.076*	
H9C	0.2864	0.1913	−0.2201	0.076*	
C10	0.6334 (8)	0.3630 (5)	0.3534 (4)	0.0613 (12)	
H10A	0.5392	0.3513	0.2808	0.092*	
H10B	0.7265	0.4560	0.3788	0.092*	
H10C	0.5097	0.3522	0.4105	0.092*	
C11	0.8541 (11)	0.2953 (6)	0.5555 (4)	0.0948 (18)	
H11A	0.6719	0.2654	0.5637	0.142*	
H11B	0.8734	0.3962	0.5680	0.142*	
H11C	0.9643	0.2713	0.6116	0.142*	

### Atomic displacement parameters (Å^2^)

**Table:**

	*U*^11^	*U*^22^	*U*^33^	*U*^12^	*U*^13^	*U*^23^
S1	0.0475 (6)	0.0432 (6)	0.0496 (6)	−0.0111 (4)	−0.0123 (4)	0.0231 (5)
O1	0.0659 (18)	0.0452 (16)	0.0549 (17)	−0.0173 (14)	−0.0174 (14)	0.0224 (14)
N1	0.050 (2)	0.0423 (19)	0.0379 (18)	−0.0132 (15)	−0.0127 (15)	0.0177 (15)
N2	0.0417 (18)	0.0414 (19)	0.0363 (17)	−0.0026 (15)	−0.0002 (14)	0.0210 (14)
C1	0.048 (2)	0.043 (2)	0.044 (2)	−0.0116 (19)	−0.0029 (18)	0.0217 (19)
C2	0.038 (2)	0.051 (3)	0.055 (3)	−0.0107 (19)	0.0014 (19)	0.021 (2)
C3	0.061 (3)	0.071 (3)	0.045 (2)	−0.0172 (17)	−0.0050 (19)	0.025 (2)
C4	0.071 (3)	0.072 (3)	0.064 (2)	−0.0141 (18)	−0.018 (2)	0.041 (2)
C5	0.079 (3)	0.061 (3)	0.084 (3)	0.009 (2)	−0.007 (3)	0.035 (2)
C6	0.057 (3)	0.050 (3)	0.063 (3)	0.001 (2)	−0.005 (2)	0.030 (2)
C7	0.040 (2)	0.035 (2)	0.034 (2)	0.0021 (16)	0.0001 (16)	0.0136 (17)
C8	0.041 (2)	0.038 (2)	0.036 (2)	0.0006 (18)	0.0001 (17)	0.0097 (17)
C9	0.053 (2)	0.055 (3)	0.047 (2)	−0.001 (2)	−0.0118 (19)	0.020 (2)
C10	0.060 (3)	0.068 (3)	0.052 (3)	0.002 (2)	0.010 (2)	0.012 (2)
C11	0.108 (4)	0.120 (5)	0.053 (3)	−0.017 (4)	−0.006 (3)	0.029 (3)

### Geometric parameters (Å, º)

**Table:**

S1—C7	1.671 (4)	C4—H4	0.9300
O1—C8	1.221 (4)	C5—C6	1.374 (6)
N1—C7	1.316 (4)	C5—H5	0.9300
N1—C1	1.440 (4)	C6—H6	0.9300
N1—H1N	0.856 (18)	C8—C9	1.484 (5)
N2—C8	1.375 (4)	C9—H9A	0.9600
N2—C7	1.383 (4)	C9—H9B	0.9600
N2—H2N	0.838 (18)	C9—H9C	0.9600
C1—C2	1.376 (5)	C10—H10A	0.9600
C1—C6	1.391 (5)	C10—H10B	0.9600
C2—C3	1.429 (5)	C10—H10C	0.9600
C2—C10	1.470 (5)	C11—H11A	0.9600
C3—C4	1.366 (6)	C11—H11B	0.9600
C3—C11	1.481 (6)	C11—H11C	0.9600
C4—C5	1.380 (7)		
			
C7—N1—C1	124.6 (3)	N1—C7—N2	116.9 (3)
C7—N1—H1N	115 (3)	N1—C7—S1	123.6 (3)
C1—N1—H1N	120 (3)	N2—C7—S1	119.5 (3)
C8—N2—C7	129.1 (3)	O1—C8—N2	121.9 (3)
C8—N2—H2N	113 (3)	O1—C8—C9	122.9 (3)
C7—N2—H2N	118 (3)	N2—C8—C9	115.2 (3)
C2—C1—C6	124.1 (4)	C8—C9—H9A	109.5
C2—C1—N1	118.7 (4)	C8—C9—H9B	109.5
C6—C1—N1	117.1 (4)	H9A—C9—H9B	109.5
C1—C2—C3	117.0 (4)	C8—C9—H9C	109.5
C1—C2—C10	122.6 (4)	H9A—C9—H9C	109.5
C3—C2—C10	120.4 (4)	H9B—C9—H9C	109.5
C4—C3—C2	118.4 (4)	C2—C10—H10A	109.5
C4—C3—C11	121.1 (4)	C2—C10—H10B	109.5
C2—C3—C11	120.5 (5)	H10A—C10—H10B	109.5
C3—C4—C5	123.1 (4)	C2—C10—H10C	109.5
C3—C4—H4	118.4	H10A—C10—H10C	109.5
C5—C4—H4	118.4	H10B—C10—H10C	109.5
C6—C5—C4	119.7 (5)	C3—C11—H11A	109.5
C6—C5—H5	120.2	C3—C11—H11B	109.5
C4—C5—H5	120.2	H11A—C11—H11B	109.5
C5—C6—C1	117.7 (4)	C3—C11—H11C	109.5
C5—C6—H6	121.2	H11A—C11—H11C	109.5
C1—C6—H6	121.2	H11B—C11—H11C	109.5
			
C7—N1—C1—C2	83.6 (5)	C11—C3—C4—C5	179.5 (4)
C7—N1—C1—C6	−99.9 (4)	C3—C4—C5—C6	−0.6 (7)
C6—C1—C2—C3	−0.4 (5)	C4—C5—C6—C1	0.8 (6)
N1—C1—C2—C3	175.8 (3)	C2—C1—C6—C5	−0.3 (6)
C6—C1—C2—C10	179.3 (3)	N1—C1—C6—C5	−176.6 (4)
N1—C1—C2—C10	−4.4 (5)	C1—N1—C7—N2	179.8 (3)
C1—C2—C3—C4	0.7 (6)	C1—N1—C7—S1	−0.1 (5)
C10—C2—C3—C4	−179.1 (4)	C8—N2—C7—N1	2.3 (6)
C1—C2—C3—C11	−179.0 (4)	C8—N2—C7—S1	−177.8 (3)
C10—C2—C3—C11	1.2 (6)	C7—N2—C8—O1	−5.1 (6)
C2—C3—C4—C5	−0.2 (7)	C7—N2—C8—C9	174.4 (3)

### Hydrogen-bond geometry (Å, º)

**Table:**

*D*—H···*A*	*D*—H	H···*A*	*D*···*A*	*D*—H···*A*
N1—H1*N*···O1	0.86 (2)	1.97 (3)	2.664 (4)	137 (3)
N1—H1*N*···O1^i^	0.86 (2)	2.50 (3)	3.168 (4)	136 (3)
N2—H2*N*···S1^ii^	0.84 (2)	2.54 (2)	3.378 (3)	176 (3)

Symmetry codes: (i) −*x*+1, −*y*, −*z*; (ii) −*x*+2, −*y*+1, −*z*.
